# Association between arterial stiffness, stress and depression among undergraduate medical students in India

**DOI:** 10.6026/973206300214934

**Published:** 2025-12-15

**Authors:** A. Logesh Priyan, Shobha M.V, Jagadamba Aswathappa, Ashwini K Shetty, C. Rutha Sneha

**Affiliations:** 1 Department of Physiology, Sri Devraj urs Academy of Higher Education & Research, Tamaka, Kolar, Karnataka, India; 2 Department of Physchiatry, Sri Devraj urs Academy of Higher Education & Research, Tamaka, Kolar, Karnataka, India

**Keywords:** Arterial stiffness, pulse wave velocity, depression, stress, medical students, cardiovascular risk

## Abstract

Arterial stiffness independently predicts cardiovascular events, with psychological factors potentially contributing to early vascular alterations in young populations. Therefore, it is of interest to investigate of 153 undergraduate medical students to examine relationships between psychological stress, depression and arterial stiffness measures (brachial-ankle pulse wave velocity, carotid-femoral pulse wave velocity, brachial-ankle stiffness index and ankle-brachial index) assessed via Beck's Depression Inventory and Perceived Stress Scale. Our findings revealed mild depression in 92.2% and mild stress in 83.7% of participants, demonstrating significant associations between depression scores and average baPWV (r=0.028, p=0.030), CFPWV (r=0.030, p=0.015), average BASI (r=0.085, p=0.029) and average ABI (r=0.333, p<0.001). Thus, we show that psychological screening tools may function as early cardiovascular risk markers, supporting implementation of comprehensive mental health and cardiovascular prevention initiatives for medical students.

## Background:

Cardiovascular disease represents the primary cause of global morbidity and mortality, with arterial stiffness serving as an independent predictor of cardiovascular events and overall mortality [1]. This condition, characterized by diminished arterial compliance and elevated pulse wave velocity, indicates structural and functional arterial wall modifications [2]. Evidence increasingly suggests that arterial stiffening commences earlier in life than previously recognized, with psychological factors including stress and depression playing potential contributory roles [3]. Chronic psychological stress triggers activation of the sympathetic nervous system, hypothalamic-pituitary-adrenal axis and inflammatory cascades, contributing to vascular dysfunction and accelerated arterial stiffening [4]. Depression correlates with elevated cardiovascular risk through endothelial dysfunction, enhanced platelet aggregation and disrupted autonomic nervous system regulation [5]. Modern non-invasive assessment technologies facilitate arterial stiffness detection through pulse wave analysis and brachial-ankle pulse wave velocity evaluation [6]. These methodologies identify subclinical vascular alterations that may emerge years before clinical cardiovascular disease manifestation [7]. Medical students encounter substantial academic, clinical and personal pressures throughout their training [8]. Limited investigation has examined how these psychological stressors influence early-stage vascular modifications in this demographic [9]. Research has investigated mental health and arterial stiffness relationships in middle-aged populations, [10] yet a knowledge deficit persists regarding young adults in high-stress educational contexts. Integrated mental health and cardiovascular prevention approaches are essential for this vulnerable population [5]. Therefore, it is of interest to establish associations between psychological stress, depression and arterial stiffness parameters among undergraduate medical students.

## Materials and Methods:

## Study design:

We conducted a cross-sectional observational study among undergraduate medical students at Sri Devaraj Urs Medical College, Kolar. Written informed consent was obtained from all participants after institutional ethical clearance.

## Sample size:

The estimated sample size comprised 153 subjects calculated based on correlation coefficient between stress parameters and arterial stiffness (r = 0.225, p = 0.005) [11].

## Study population:

## Inclusion criteria:

Undergraduate medical students aged 18-25 years, both males and females.

## Exclusion criteria:

Subjects with history of sleep disorders, psychological disorders, neurological and endocrine disorders.

## Data collection:

We collected data through self-administered questionnaires comprising three sections. The initial section documented demographic and anthropometric measurements: age, gender, education level and physical activity status. Anthropometric assessments included weight and height measurements, with subsequent Body Mass Index calculation.

## Perceived Stress Scale (PSS):

This self-report instrument evaluates the extent to which individuals perceive life circumstances as unpredictable, uncontrollable and overwhelming [12]. Scoring: 0-13 represents low stress, 14-26 indicates moderate stress and 27-40 signifies high perceived stress.

## Beck's depression inventory:

This validated self-assessment tool comprises 21 items scored on a 0-3 Likert scale [13]. Scoring: 0-13 represents minimal depression, 14-19 indicates mild depression, 20-28 suggests moderate depression and 29-63 signifies severe depression.

## Blood pressure assessment:

We obtained three consecutive brachial blood pressure measurements with participants seated after 5 minutes of rest, maintaining arm position at heart level. We subsequently calculated mean arterial blood pressure and pulse pressure.

## Pulse wave velocity assessment:

We performed measurements using the PeriScope® device (Genesis Medical Systems Pvt. Ltd., Hyderabad, India), which employs oscillometric methodology for non-invasive arterial pressure waveform capture. The system recorded ECG-gated pressure waveforms simultaneously from bilateral arms and ankles. We conducted procedures during morning hours following 10-hour overnight fasting. Participants abstained from smoking for minimum 4 hours pre-procedure. After 10 minutes in supine position, we applied four blood pressure cuffs on bilateral arms and ankles. We positioned ECG electrodes on wrists and ankles for simultaneous ECG recording. The system automatically inflated and deflated cuffs while recording pressure waveforms. We calculated bilateral brachial-ankle PWV (baPWV) from these waveforms for analysis.

## Statistical analysis:

We entered and analysed data using IBM SPSS Statistics Version 26.0 (IBM Corp., Armonk, NY, USA). We presented descriptive statistics as mean ± standard deviation for continuous variables and as frequencies with percentages for categorical variables. We applied Pearson's correlation coefficient to examine associations between depression/stress scores and arterial stiffness parameters. We performed group comparisons using independent samples t-test. We conducted multiple linear regression analysis to identify independent arterial stiffness predictors, entering age, blood pressure, depression and stress scores as covariates. We considered p-values < 0.05 as statistically significant.

## Results:

Our study included 153 undergraduate medical students. Mean participant age was 24.17 ± 9.13 years, with female predominance (64.1%). Mean BMI measured 23.96 ± 4.19 kg/m^2^. Cardiovascular parameters fell within normal ranges: mean systolic blood pressure 115.95 ± 13.18 mmHg and mean diastolic blood pressure 73.36 ± 8.50 mmHg. Beck's Depression Inventory results showed 92.2% of students reported mild depression, while 7.8% exhibited no depression. Perceived Stress Scale results indicated 83.7% reported mild stress and 16.3% reported moderate stress. Depression scores demonstrated significant correlations with average baPWV (r = 0.028, p = 0.030), CFPWV (r = 0.030, p = 0.015), average BASI (r = 0.085, p = 0.029) and average ABI (r = 0.333, p < 0.001). Stress scores showed significant correlations with average baPWV (r = 0.079, p = 0.033), CFPWV (r = 0.105, p = 0.019) and average BASI (r = 0.147, p = 0.040) (Table 1 - see PDF). Students with mild depression had significantly elevated CFPWV (p = 0.013), elevated BASI (p = 0.024) and reduced ABI (p < 0.001) versus non-depressed students. Students with moderate stress had significantly elevated BASI (p = 0.016), elevated systolic BP (p = 0.020) and elevated diastolic BP (p = 0.010) compared to mild stress group. Multiple linear regression analysis identified CFPWV (β = 0.959, p < 0.001), age (β = 0.092, p < 0.001) and systolic blood pressure (β = 0.167, p < 0.001) as independent arterial stiffness predictors. Depression and stress scores did not emerge as independent predictors after controlling for other variables (Table 2 - see PDF).

## Discussion:

This cross-sectional investigation reveals significant correlations between depression scores, perceived stress and arterial stiffness parameters in young medical students. Our findings demonstrate that 92.2% of participants exhibited mild depression, with 83.7% experiencing mild stress levels. This substantial prevalence underscores the necessity for targeted mental health interventions within medical education settings [14]. Observed mean bilateral brachial-ankle PWV (1198.04 ± 261.95 cm/s) and carotid-femoral PWV (761.49 ± 223.24 cm/s) align with reported values for healthy young adults. Ankle-brachial index values (mean 1.03 ± 0.07) remained within normal parameters, indicating preserved peripheral vascular function. Substantial standard deviations suggest considerable inter-individual variation, potentially reflecting psychological factor influences on vascular health. Depression scores correlated significantly with multiple arterial stiffness parameters. The strongest correlation emerged with ABI (r=0.333, p<0.001), suggesting depression may particularly affect peripheral vascular function. These findings align with recent research demonstrating psychological factor influences on vascular function across different populations [15]. Depression activates inflammatory pathways, increases sympathetic nervous system activity and promotes endothelial dysfunction-all mechanisms contributing to arterial stiffening. Students with mild depression demonstrated differential effects on central versus peripheral arterial stiffness measures. While CFPWV represents the gold standard for central arterial stiffness assessment, baPWV reflects combined central and peripheral arterial properties. These differential effects suggest depression may influence central and peripheral arteries through distinct mechanisms, possibly related to variations in sympathetic innervation and local inflammatory responses. Perceived stress scores correlated significantly with arterial stiffness parameters. These findings support recent research demonstrating mental stress augmentation of central artery stiffness in young individuals. The physiological basis involves stress-induced activation of hypothalamic-pituitary-adrenal axis and sympathetic nervous system, leading to increased vascular tone and accelerated arterial stiffening. Participants with moderate stress exhibited elevated BASI scores (27.9 ± 6.44 versus 25.0 ± 5.13, p=0.016) and higher blood pressure levels compared to mild stress group. While statistically significant, blood pressure differences remained within normal ranges, suggesting stress-related vascular changes may precede clinically apparent hypertension. These findings align with reported associations between acute psychological stress, autonomic function and arterial stiffness among women [16]. Regression analysis revealed that psychological parameters did not emerge as significant independent predictors when controlling for other variables. Strongest predictors included CFPWV (β=0.959, p<0.001), age (β=0.092, p<0.001) and systolic blood pressure (β=0.167, p<0.001). This suggests psychological effects on arterial stiffness may be mediated through hemodynamic parameters. The absence of psychological parameters as independent predictors highlights complex interrelationships between psychological stress, blood pressure and vascular function. Psychological screening tools including BDI and PSS could function as early cardiovascular risk indicators. The robust association between depression and ABI suggests simple, non-invasive vascular assessments could complement psychological evaluations for identifying students at future cardiovascular disease risk. This approach aligns with emerging concepts of integrated cardiovascular risk assessment considering both traditional and psychological risk factors. Our findings align with reported associations between depressive symptoms, perceived stress and arterial health in adolescents. Similarly, investigations identified associations in middle-aged adults, indicating persistence across age groups. Our results extend previous findings in medical staff to medical students, representing earlier career stages when interventions may prove most effective. Observed moderate correlation coefficients align with literature, suggesting psychological factors represent one component of multifactorial processes influencing vascular health [17, 18]. This investigation employed validated psychological assessment instruments and multiple arterial stiffness parameters, providing comprehensive vascular function evaluation. Focus on medical students represents a unique vulnerable population experiencing specific stressors. The cross-sectional design enabled association examination without potential confounding from longitudinal health status changes. The 153-participant sample size provides adequate power for detecting moderate effect sizes.

## Conclusion:

This investigation demonstrates significant associations between psychological parameters and arterial stiffness in young medical students, highlighting a notable burden of psychological distress with measurable effects on vascular function. Although causality cannot be established, the findings suggest that psychological screening tools may serve as early indicators of cardiovascular risk, with stress-related vascular changes beginning at a younger age than previously recognized. Longitudinal and interventional studies are warranted to confirm causality and to evaluate whether stress-reduction interventions can improve both psychological well-being and cardiovascular health in young adults.
